# Codelivery of Paclitaxel and Cannabidiol in Lipid Nanoparticles Enhances Cytotoxicity against Melanoma Cells

**DOI:** 10.1021/acsomega.5c00689

**Published:** 2025-05-22

**Authors:** Fabíola V. de Carvalho, Gabriela Geronimo, Ludmilla D. de Moura, Talita C. Mendonça, Márcia Cristina Breitkreitz, Eneida de Paula, Gustavo H. Rodrigues da Silva

**Affiliations:** † Department of Biochemistry and Tissue Biology, Institute of Biology, University of Campinas (UNICAMP), 13083-862 Campinas, São Paulo, Brazil; ‡ Brazilian Biosciences National Laboratory, Brazilian Center for Research in Energy and Materials, 13083-100 Campinas, São Paulo, Brazil; § Department of Analytical Chemistry, Institute of Chemistry, University of Campinas (UNICAMP), 13083-970 Campinas, São Paulo, Brazil

## Abstract

Although chemotherapy regimens are well-established in clinical practice, chemoresistance and adverse side effects pose significant obstacles in cancer treatment. Paclitaxel (PTX), a widely used chemotherapeutic agent, faces formulation challenges due to its poor solubility and permeability. Research suggests that the phytochemical Cannabidiol (CBD) holds potential not only in targeting cancer cells but also in alleviating pain and nausea, thereby improving the quality of life for cancer patients. However, CBD’s clinical application is also limited by its poor solubility, low bioavailability, and susceptibility to oxidation. Nanostructured lipid carriers (NLCs) represent a promising drug delivery system for hydrophobic compounds like PTX and CBD and allow their coencapsulation. Nonetheless, achieving a stable formulation requires the identification of suitable preparation methods and excipients. The aim of this study was to develop and optimize an NLC formulation for the coencapsulation of PTX and CBD. Using factorial design, an optimized formulation was obtained with homogeneous particle sizes (200 nm), negative ZPs (−16.1 mV), a particle concentration of 10^13^ particles/mL, spherical morphology (TEM images), and a lipid core with low crystallinity (confirmed by XRD). To evaluate the therapeutic potential of the drug combination, cell viability assays were conducted on murine melanoma cells (B16–F10) at different exposure times (24 and 48 h). The NLC–CBD-PTX formulation significantly reduced cell viability in a time- and concentration-dependent manner, demonstrating at least 75% greater activity at 24 h compared to each drug individually, whether free (PTX, CBD) or encapsulated (NLC-PTX, NLC–CBD). This indicates a synergistic effect between CBD and PTX when coencapsulated, particularly at higher concentrations and shorter exposure times. In conclusion, an innovative pharmaceutical formulation coencapsulating PTX and CBD was validated, showing potential to enhance antitumor efficacy, overcome chemoresistance, reduce side effects, and broaden therapeutic applications. The resulting NLCs exhibited favorable physicochemical properties, supporting their suitability for various routes of administration.

## Graphical abstract

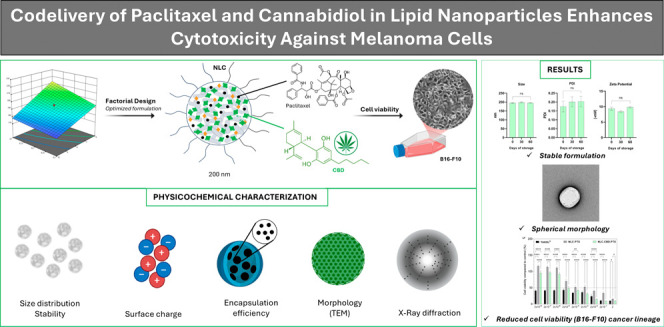

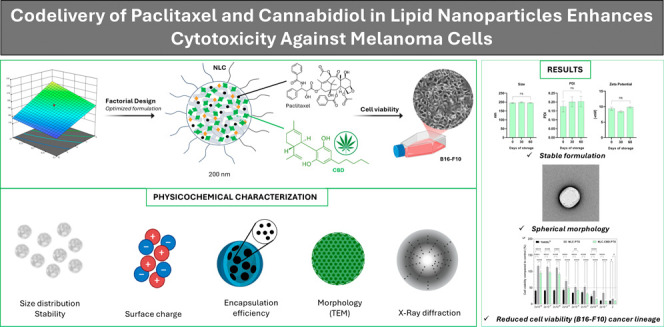

## 1 Introduction

Melanoma is one of the most serious types of skin cancer, characterized by its high frequency of metastasis, which makes it more dangerous than other forms of skin cancer.^1^ In 2022, an estimated 330,000 new cases of melanoma were diagnosed worldwide, resulting in nearly 60,000 deaths. The incidence of melanoma varies significantly between countries and regions, with higher rates seen in men than in women in most areas.^2^ In the therapeutic context, the National Comprehensive Cancer Network (NCCN) guidelines indicate that although paclitaxel is not a first-line treatment, it can be used in certain combination chemotherapy regimens, especially for advanced or resistant melanomas.^3,4^

Chemotherapy treatment protocols are well-established in clinical practice. PTX is a broad-spectrum chemotherapeutic agent approved by the Food and Drug Administration (FDA) in 1992 for the treatment of various types of cancer, from early to advanced stages.^5,6^ Derived from the plant Taxus brevifolia, PTX belongs to taxane class antineoplastics and exhibits cytotoxic activity against many solid tumors.^7^ By binding to the N-terminal amino acids of the tubulin β-subunit, PTX stabilizes the main protein of microtubules and enhances its polymerization, disrupting mitosis in the G2/M phase and leading to cell death.^7,8^ However, being a class IV drug in the Biopharmaceutics Classification System, PTX has low aqueous solubility and permeability.^5,7,9–11^ Commercial pharmaceutical formulations for intravenous administration, such as TAXOL, contain noninert excipients (ethanol and polyoxyethylated castor oil) that increase PTX toxicity, resulting in severe side effects such as cardiovascular issues, neutropenia, and central or peripheral neurotoxicity, limiting its clinical applicability.^12–14^ In the context of chemotherapy, encapsulation in drug delivery systems (DDS) can minimize these undesirable effects by providing sustained drug release to avoid plasma spikes and targeting the drug to tumor cells.^15^

Cannabidiol (CBD) has been extensively studied for the treatment of various conditions and diseases such as chronic pain, inflammation, and cancer.^16–22^ A recent review highlighted the effects of CBD on human cancer cells from various tissues, including gastrointestinal, genital, mammary, respiratory, nervous, hematopoietic, and skeletal systems, showing a decrease in cell viability, proliferation, migration, inflammation, and metastasis.^16^ In the case of melanoma, recent studies have demonstrated the potential antineoplastic effects of CBD through various molecular mechanisms, primarily involving the reduction of tumor cell viability, proliferation, migration, and angiogenesis, as well as the induction of apoptosis in preclinical models.^23–25^ Despite the numerous pharmaceutical activities attributed to CBD, its clinical applicability is hindered by low aqueous solubility (0.1 μg/mL), limited bioavailability (6–12%; oral route^26^), and susceptibility to oxidation,^27,28^ demanding the development of strategies to enhance its clinical use.^29^

Drug delivery systems (DDS) have gained increasing importance in the research and development of new medications due to their numerous advantages. These include protection of the drug from enzymatic degradation, prolonged circulation time in the bloodstream, reduced therapeutic doses, ease of administration, and lower toxicity with increased bioavailability. Additionally, their small size allows for greater accumulation in target tissues, enhancing therapeutic efficacy.^30^ Nanostructured lipid carriers (NLC) are lipid nanocarriers composed of a lipid core (a mixture of solid and liquid lipids at room/body temperature) stabilized by a surfactant. The lipid blend makes the core less ordered, allowing for higher drug upload and minimizing drug expulsion during storage.^31,32^

Chemoresistance poses a significant challenge in the effective treatment of cancer, as tumor cells develop resistance to chemotherapy drugs.^33,34^ DDS may overcome drug resistance, enhancing drug delivery and improving treatment efficacy.^23,35^ Indeed, when it comes to encapsulating chemotherapy drugs in NLCs, they have shown to surpass the limitations of traditional chemotherapy by enhancing drug loading capacity, stability, targeted delivery, and chemoresistance.^36,37^ Additionally, NLCs can be tailored to coencapsulate multiple therapeutic agents, such as chemotherapeutic drugs and genetic material, metabolic modulators, or siRNA, to achieve synergistic effects and combat drug resistance.^38,39^

Despite scientific advancements in the field, the development of more efficient antitumor formulations that are selective to the target cell, are water-soluble for parenteral application, and have low systemic toxicity remains a significant challenge. Therefore, the aim of this study was to combine PTX and CBD in NLCs to attain enhanced anticancer properties and reduce side effects such as PTX-induced peripheral neuropathy. Additionally, cannabinoids may contribute to a better quality of life for cancer patients, offering palliative effects such as pain relief and nausea reduction.^40^ In this study, we developed an optimized NLC–CBD-PTX formulation using experimental design, characterized it through techniques such as DLS (dynamic light scattering), NTA (nanoparticle tracking analysis), TEM (transmission electron microscopy), and XRD (X-ray diffraction), and evaluated its stability. Finally, we assessed the cytotoxicity of the coencapsulated actives in melanoma cells to determine their efficacy in antitumor activity.

## 2 Materials and Methods

### 2.1 Materials

Paclitaxel powder (PTX) was a gift from Cristália Prod. Quim. Farm. Ltda (São Paulo, SP, Brazil), and myristyl myristate (MM) was donated by Croda do Brasil Ltda (Campinas, SP, Brazil). Commercial PTX (TAXOL) was purchased in the Brazilian market. Cannabidiol oil (200 mg/mL) was purchased from Prati-Donaduzzi & Cia Ltda (Toledo, PR, Brazil). Soy L-α-phosphatidylcholine (SPC) was obtained from Avanti Polar Lipids, Inc. (Alabaster, AL, USA). Pluronic F-68 (P68), Dulbecco’s Modified Eagle Medium (DMEM), fetal bovine serum (FBS), 3-(4,5-dimethylthiazol-2-yl)-2,5-diphenyltetrazolium bromide (MTT), penicillin, streptomycin sulfate, and trypsin were supplied by Sigma Chem. Co. (St. Louis, MO, USA). Dimethyl sulfoxide (DMSO) was purchased from Laborclin (Pinhais, PR, Brazil) and HPLC-grade methanol from J.T. Baker (Allentown, PA, USA). Melanoma cells (B16–F10 cell line) were purchased from the American Type Culture Collection (ATCC, Manassas, VA, USA). Deionized water (18 MΩ) was obtained with an Elga USF Maxima ultrapure water purifier.

### 2.2 Methods

#### 2.2.1 NLC Preparation

NLCs were prepared using a modified emulsification–ultrasonication technique.^41^ Initially, PTX, CBD, or both actives were dissolved in the lipids at a temperature 10 °C above the solid lipid’s melting point, with the addition of ethanol, and subjected to 10 min of heating and mechanical agitation in a water bath. Simultaneously, a surfactant solution was heated to the same temperature, and both phases were mixed at high speed (10,000 rpm) for 3 min using an Ultra-Turrax blender (IKA Werke, Staufen, Germany). Subsequently, the mixture underwent 16 min of sonication in a Vibracell tip sonicator (Sonics & Materials Inc., Danbury, USA) operating at 500 W and 20 kHz, in alternating 30-s cycles. The resulting nanoemulsion was cooled to room temperature to form the NLC. All prepared formulations remained as liquid suspensions and were stored at room temperature (25 °C) for the next tests.

#### 2.2.2 Composition Optimization by Experimental Design

The excipients were selected based on their widespread use in the development of nanostructured lipid carriers (NLCs)^42,43^ and the group’s previous experience with hydrophobic taxanos.^44,45^ The choice of excipient proportions was guided through factorial design using Design Expert software (version 13, Stat-Ease, Inc., Minneapolis, USA), ensuring the optimization of critical formulation parameters.^46,47^ A full factorial design 2^3^ was developed with triplicates at the central point. Three independent variables were evaluated at two levels (high and low). The maximum and minimum factor levels were determined through preliminary screening tests, taking into consideration previous design experiences of the group. Table 1 displays the design conditions, with fixed quantities of 1g of cannabidiol oil (200 mg/mL) and 60 mg of PTX. The criteria for optimizing included minimum particle size (versatility of application), minimal PDI (monodisperse systems), and higher ZP values in module.^48,49^ The validation of the mathematical models was performed through analysis of variance (ANOVA, Tables S1–S3 of the Supporting Information), prediction capacity (Figure S2), and analysis of residuals (not shown).

**Table 1:** Design of Experiments^a^

experimental variables	symbol in the program	low level	high level
myristyl myristate (%)	A	8	12
L-α-phosphatidylcholine (g)	B	0.1	0.3
pluronic F-68 (%)	C	6	10

properties of interest	criteria for optimization
size (nm)	minimum
PDI	minimum
ZP (mV)	maximum

a Experimental variables, levels, properties of interest (responses), and criteria for optimizing. Average diameters (size), polydispersity index (PDI), and ζ potential (ZP).

#### 2.2.3 Physicochemical Characterization of NLC

##### 2.2.3.1 Measurement of the Particle Size and Polydispersity Index

The average diameter and PDI of the NLCs were measured by DLS. Dilutions of the different suspensions in deionized water (1000x) were measured in triplicate using the ZetaSizer Nano ZS90 equipment (Malvern Instruments, Malvern, Worcestershire, UK) at 25 °C, coupled to a data acquisition system.

##### 2.2.3.2 ζ Potential Measurement

ZP values were determined by Laser Doppler Microelectrophoresis using the ZetaSizer Nano ZS90 equipment (Malvern Instruments, UK). Measurements were performed in triplicate in appropriate polystyrene cuvettes, diluting the NLC suspensions in deionized water (1000×), at a temperature of 25 °C.^48^ The data were expressed as mean ± SD.

##### 2.2.3.3 HPLC Method and Determination of the Encapsulation Efficiency

The drugs PTX and CBD were quantified by high-performance liquid chromatography (HPLC) under the conditions described in Table 2. The equipment used was a Waters Breeze 2 high-performance liquid chromatograph (Waters Technol., MA, USA). The retention times for PTX and CBD were 4.9 and 20 min, respectively, (as shown in the chromatogram of Figure S1).

**Table 2:** Chromatographic Conditions for the Simultaneous Quantification of PTX and CBD in NLCs

parameters	conditions
column	C18 Gemini-NX 5 μ 450 × 4.60 mm
oven temperature	30 °C
mobile phase	acetonitrile, deionized water and acetic acid, 70:30:0.1 (v/v/v)
flow	1 mL min^–1^
injection volume	10 μL
wavelength	230 nm

The percentage of encapsulation efficiency (%EE) was determined by the ultrafiltration–centrifugation method, using cellulose filters (10 kDa, Millipore).^48,50^ For this purpose, an aliquot of the formulation was added to the filtration unit, coupled to Eppendorf tubes, and centrifuged for approximately 20 min at 4100g. The amount of free antineoplastic in the filtrate was quantified by HPLC, and the percentage of encapsulated antineoplastic was calculated according to eq 1.

$$\%\mathrm{EE}=\frac{{\mathrm{Drug}}_{\mathrm{total}}-{\mathrm{Drug}}_{\mathrm{free}}}{{\mathrm{Drug}}_{\mathrm{total}}}\times 100$$ 1

where Drug_total_ is the total amount of PTX or CBD quantified in the NLC suspension, and Drug_free_ corresponds to the amounts of PTX or CBD in the filtrate.

##### 2.2.3.4 Nanoparticle Tracking Analysis

NTA was used to determine the size, distribution, and concentration of nanoparticles in the formulation, on an NS300 instrument (NanoSight, Amesbury, UK) equipped with a 532 nm laser. Based on the tracking of individual Brownian motion of nanoparticles, NTA is the only real-time technique that provides nanoparticle concentration (number of particle/mL).^51^ Samples were diluted in deionized water (50,000x) and introduced into the sample holder using a syringe until it was filled completely. Measurements were performed at room temperature, in triplicate, with the data expressed as the mean ± SD.

The polydispersity or particle size distribution data were obtained by calculating the SPAN index, according to eq 2.^52^

$$\mathrm{SPAN}=\frac{D90\%-D10\%}{D50\%}$$ 2

where D10, D50, and D90 refer to the mean size of 10, 50, and 90% of the particle population, respectively.

##### 2.2.3.5 X-ray Diffraction

XRD measurements were carried out using a D2-Phaser diffractometer (Bruker, Germany) under the following experimental conditions: a temperature of 293 K, Cu–Kα radiation (λ = 1.5418 Å) generated at 30 kV and 10 mA, continuous scan mode with a step time of 0.2 s, and an angular range (2θ) of 5–50° with an increment of 0.02°. The NLC samples, initially in liquid form, were freeze-dried to enable XRD analysis. The generated data were processed using Origin software, version 8.2.

##### 2.2.3.6 Transmission Electron Microscopy

To analyze the morphology of the nanoparticles, a Tecnai G2 Spirit BioTWIN Transmission Electron Microscope (FEI Company, USA) with an accelerating voltage of 60 kV was used. Before staining, NLC samples were diluted 33 times in deionized water and placed on a Formvar film covered with a copper grid. Then, the samples were stained with 2% uranyl acetate. After being washed with deionized water and dried at room temperature (24 h), the grid replica was prepared in a sample holder and placed in the vacuum chamber of the instrument. NLCs containing PTX only or combined with CBD were prepared by using the same procedure. ImageJ software (version 1.53k, NIH, Bethesda, MD, USA) was used to edit the images and measure the particles size.^53^

#### 2.2.4 Evaluation of Formulation Stability

The stability of the optimized formulation and its control, stored at room temperature (23–28 °C), was evaluated for 2 months, as an aqueous dispersion. The analyzed parameters were: size (nm), PDI and ZP (mV), in addition to the visual inspection (appearance) of the formulations. Particle size, PDI, and ZP are critical for evaluating the stability of NLCs. Particle size directly influences drug stability and delivery efficiency, as larger particles tend to aggregate, compromising stability.^54^ PDI measures particle size distribution, with lower values indicating a more uniform distribution, which is essential for maintaining the stability of NLCs.^55^ Finally, ZP is a key indicator of colloidal stability; high positive or negative values ensure greater repulsion between particles, preventing aggregation and sedimentation.^56^ These parameters were chosen because they provide comprehensive insights into the physical stability and uniformity of NLC formulations.

#### 2.2.5 In Vitro Release Experiment

For the in vitro release studies, Franz-type vertical diffusion cells with a permeation area of 0.6 cm^2^ and a volume of 15 mL were utilized. The donor compartment was loaded with 0.2 mL of the following formulations: commercial paclitaxel (TAXOL, 6 mg/mL), a commercially available cannabidiol oil (20 mg/mL), and nanostructured lipid carrier-encapsulated paclitaxel and cannabidiol (NLC–CBD-PTX, with 6 mg/mL of PTX and 20 mg/mL of CBD). The receptor compartment was filled with a solution composed of 5 mM phosphate-buffered saline (PBS, pH 7.4), ethanol, and Tween 80 in a volumetric ratio of 80:15:5 (v/v)^45^ in order to keep the sink condition. A polycarbonate membrane (47 mm diameter, 100 nm molecular weight cutoff, Nucleopore Track-Etch Membrane, Whatman) separated the donor and receptor compartments. The receptor solution was maintained under gentle agitation at 300 rpm and constant temperature (37 °C) throughout the experimental period. At predetermined time intervals, 0.2 mL aliquots were collected from the receptor compartment, with the volume immediately replenished with the PBS:ethanol:Tween 80 solution. The collected samples were subsequently analyzed for paclitaxel and cannabidiol content via HPLC. The release curves were analyzed using the KinetDS 3.0 software.^57^ Several mathematical models, including zero-order, first-order, Korsmeyer–Peppas, and Weibull models, were evaluated. The best fit for the NLC release curves was found with the Weibull model,^58^ based on the coefficient of determination (*R* ^2^). The Weibull model^58^ is represented by eq 3:

$$M_{t}=M_{0}[1-e^{-(\frac{t}{T})\beta }]$$ 3

where *M*_*t*_ is the amount of the drug released at time *t*; *M*_0_ is the total amount of the drug to be released; *T* is the time constant related to the release process (also called the scale parameter); and β is the shape parameter, which describes the shape of the release curve.

#### 2.2.6 Cell Viability Assay

##### 2.2.6.1 Cell Culture

Cells of the B16–F10 cell line (murine melanoma cells, ATCC CRL 6475) were cultured in DMEM (Dulbecco’s modified Eagle’s medium), supplemented with 10% FBS (fetal bovine serum) and 1% antibiotic (penicillin and streptomycin), and brewed in an incubator (Shel Lab–CO_2_ incubator, USA) at 37 °C with atmospheric humidity, containing 95% air and 5% CO_2_. The culture medium was changed every 2 days, and from the subconfluence of more than 70% of the flask, cell subcultures were performed with the aid of trypsin.

##### 2.2.6.2 Cell Viability Assay

96-well microplates were used for plating—at a cell density of 2 × 10^4^ cells/well (24 h) or 1 × 10^4^ cells/well (48 h)—incubated at 37 °C, with 5% CO_2_, for 24 h for cell adhesion to occur. After this period, the medium was replaced with medium containing the respective treatment groups diluted, at 9 increasing concentrations (2 to 2 × 10^–8^ mg/mL), and these treatments were performed for 24 and 48 h. After the incubation period, the supernatant was removed, the wells were washed with 5 mM PBS, and viability was assessed by the soluble MTT (3-(4,5-dimethylthiazol-2-yl)-2,5-diphenyl tetrazolium bromide) reduction test: 0.5 mg/mL of MTT was added to the plate that was kept in the absence of light for 3 h at 37 °C. After this period, the medium was carefully removed, and DMSO was added to solubilize formazan crystals (produced by the degradation of MTT by the action of mitochondrial dehydrogenases of viable cells).^59^ Finally, the plates were shaken for 10 min and the absorbance corresponding to each well was read in a ELx800-GEN5RC Elisa plate reader (Life Res. Co. London, England) at λ = 570 nm. The values were expressed as a percentage of MTT reduction in relation to the control, in which the cells were not exposed to the treatment.

##### 2.2.6.3 Combined Drug Effect Analysis

In order to determine whether the effect of the combination of the compounds on the cell viability assay was synergistic, additive, or antagonistic, the SiCoDEA (Single and Combined Drug Effect Analysis, available at: https://sicodea.shinyapps.io/shiny/) program^60^ was utilized.

## 3 Results and Discussion

### 3.1 Composition Optimization by the Experimental Design

The initial step in formulating the NLCs involved identifying the optimal lipid blend for the encapsulation of the active ingredients. The solid lipid myristyl myristate was selected based on previous research conducted by the group with another taxane drug.^44^ Similarly, the nonionic surfactant Pluronic F-68 was chosen. As for the liquid lipid, we picked a commercial cannabidiol oil prepared in corn oil. To enhance the system’s stability, we incorporated SPC as a cosurfactant, commonly used in NLCs designed for antitumor agents.^37^ A modification in the traditional preparation method of NLCs through ultrahomogenization and sonication was the approach taken for solubilizing paclitaxel in the lipid phase, with a small amount of ethanol.

Although the lipid composition was predetermined, we employed experimental design to investigate the influence of each excipient on the desired properties of the formulation, including particle physical properties such as size, PDI, and ZP. Additionally, we analyzed how variations in these factors affected the final visual characteristics of the formulation (e.g., liquid formulation, highly viscous formulation such as a gel, or the formation of precipitates) after 1 week of preparation. As a result, nine different formulation compositions were studied (eight from the experimental design plus one of the central points, prepared in triplicate). The results of the experimental design are presented in Table 3.

**Table 3:** Results Obtained from the 2^3^-Factorial Design, Showing the Independent Variables (MM, SPC, and P68) and the Dependent Variables (Size, PDI, and ZP) for the NLC–CBD-PTX Development

	factor 1	factor 2	factor 3	response 1	response 2	response 3
formulation	A: MM (%)	B: SPC (g)	C: P68 (%)	size (nm)	PDI	ZP (mV)
**1**	8	0.1	6	226.7	0.195	–3.9
**2**	12	0.1	6	199.1	0.193	–8.5
**3**	8	0.3	6	191.7	0.186	–11.7
**4**	12	0.3	6	195.6	0.164	–10.3
**5**	8	0.1	10	181.1	0.248	–6.8
**6**	12	0.1	10	174.7	0.195	–2.7
**7**	8	0.3	10	172.6	0.306	–10.0
**8**	12	0.3	10	173.4	0.232	–5.9
**9**	10	0.2	8	201.7	0.169	–16.1
**10**	10	0.2	8	207.2	0.206	–13.1
**11**	10	0.2	8	195.0	0.175	–9.3

Regarding the influence of excipient variation on the physical properties of particles, the parameters of size, PDI, and ZP values of the formulations were analyzed immediately after preparation. The particle sizes ranged from 172.6 to 226.7 nm. The mathematical model generated was significant and well-fitted (ANOVA, Table S1). As expected, SPC and P68 had negative effects on size (Figure 1A and Table 4), meaning that higher concentrations resulted in smaller-sized NLCs. This can be explained based on the fact that surfactants and cosurfactants act to reduce the surface tension between the aqueous and lipid phases, leading to smaller particles.^61^

**Figure 1:**
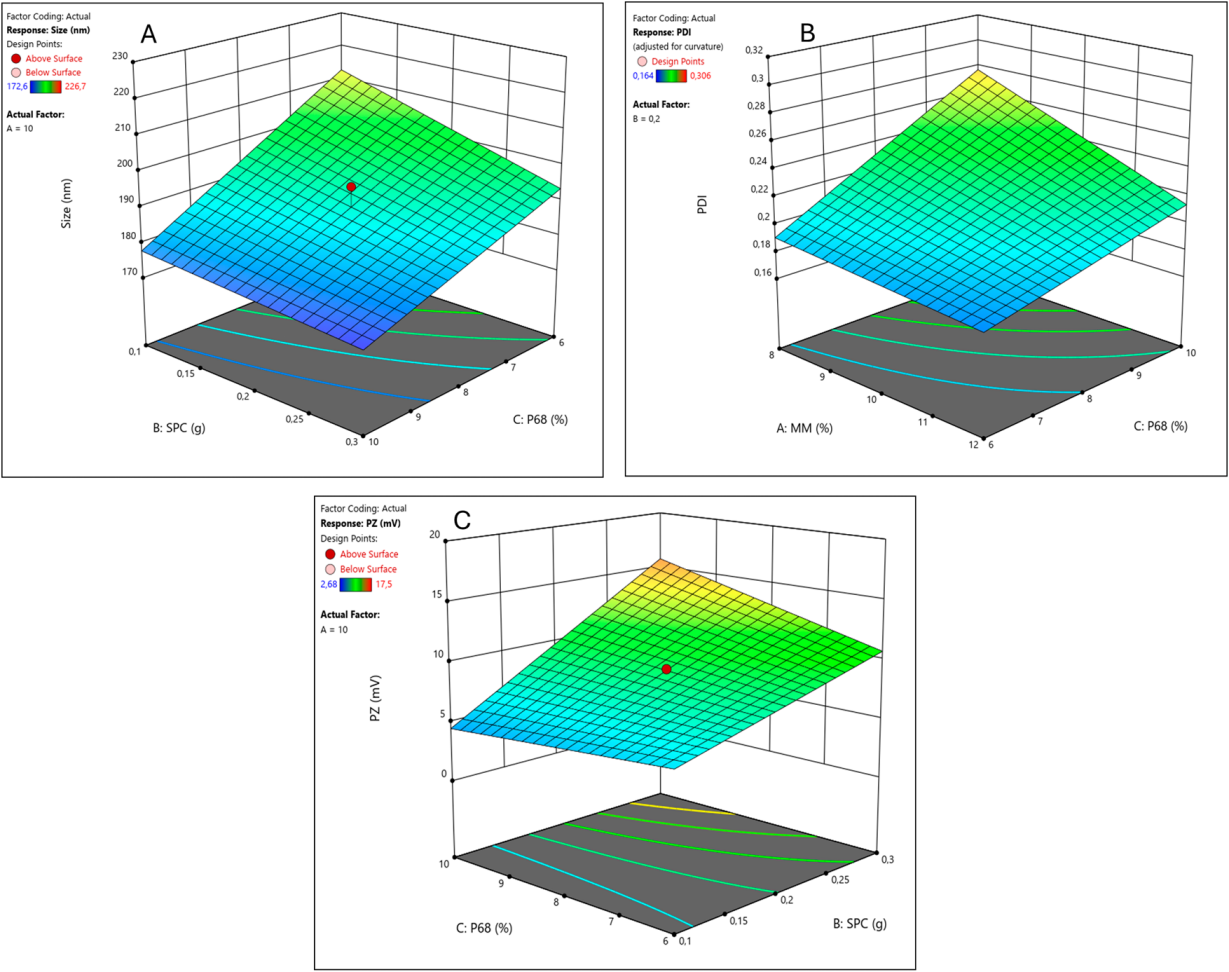
Factorial design results for the NLC–CBD-PTX system: response surfaces for size (A), PDI (B), and ZP (C).

**Table 4:** Components with Significant Effects and Their Interactions in Each Response Analyzed for the NLC–CBD-PTX Formulation

response	positive effect	negative effect
size		B (SPC), C (P68)
PDI	C (P68), BC (SPC + P68)	A (MM), AC (MM + P68)
ZP	B (SPC), BC (SPC + P68), ABC (MM + SPC + P68)	-

PDI values ranged from 0.164 to 0.306, with four samples showing a PDI above 0.2 (samples 5, 7, 8, and 10), indicating polydisperse nanoparticles.^42^ The mathematical model obtained was significant but required adjustment; therefore, the curvature term was added (see Table S2). The interactions between surfactant/cosurfactant and solid lipid/surfactant were significant, indicating the importance of using a multivariate method for designing the formulation (see Figure 1B and Table 4).

The ZP ranged from −2.7 to −16.1 mV. The mathematical model obtained was linear and showed no lack of fit (Table S3). Interestingly, the cosurfactant SPC had a significant positive effect on this response; in other words, the higher the amount of SPC, the higher the absolute ZP values (Figure 1C and Table 4). As a cosurfactant, this is a positive outcome as its addition to the system increased electrostatic repulsion between particles, potentially enhancing their colloidal stability. Moreover, at a higher concentration of SPC, the surfactant P68 played a crucial role in the steric stability of the formulation, essential for particle stability,^62,63^ as can be seen in Figure 1C.

The expertise of the group indicates that some formulations may exhibit structural instability after 1 week of preparation, leading to phase separation, precipitation, or other visual phenomena. Therefore, the formulations prepared during the experimental design were monitored after 1 week to assess their visual stability. It was observed that all formulations prepared with a low level of P68 (6%) showed some form of visual instability after 1 week (formulations 1 to 4, Table 3). According to the desirability graph for this experimental design (Figure S3), the optimized formulations (smaller size and PDI and higher ZP)^48^ should contain 6% of P68. However, a low P68 concentration leads to long-term instability of the formulation. Due to this factor, we have selected the optimized formulations of the central point (8% P68) as they meet the desired criteria and remain visually stable. So, formulation number 9 (10% MM, 0.2 g SPC, 1g CBD oil, 60 mg PTX, and 8% P68), belonging to the central point group, had its physical stability analyzed in terms of size, PDI, and ZP over a period of 60 days (Figure 2), and the results indicated that it remains stable for the period, with no significant changes in these parameters. In addition to the physical properties of the NLCs studied during this period, the encapsulation efficiency (%EE) of the drugs in this formulation also remained stable compared with the initial results, indicating the continued stability of the drugs within the lipid matrix.

**Figure 2:**
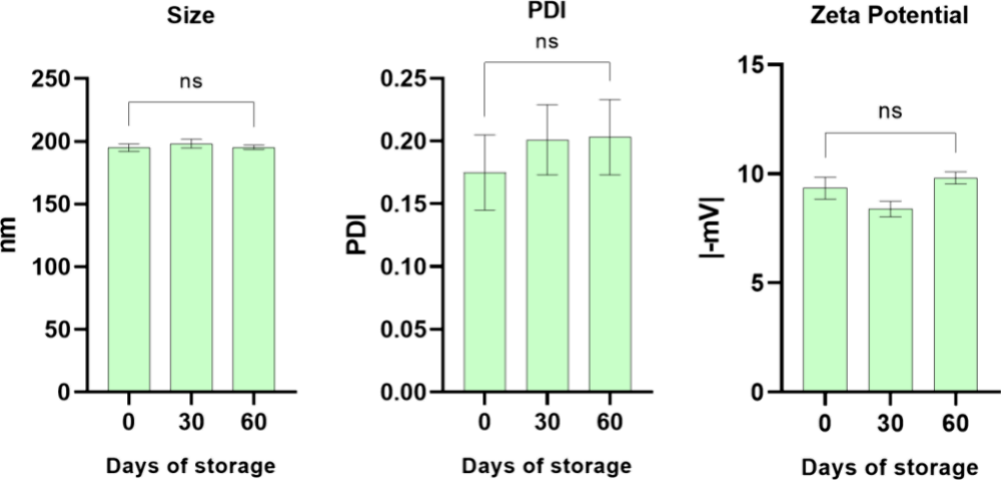
Physical stability in terms of size, PDI, and ZP of the nanoparticles of the optimized NLC (formulation 9) containing cannabidiol and paclitaxel under storage at room temperature for 60 days. ANOVA post hoc Tukey test: ns, nonsignificative.

### 3.2 Characterizations of Optimized Formulation

#### 3.2.1 DLS and Entrapment Efficiency

A fresh new batch of NLCs was prepared for the continuation of the study, based on the chosen composition determined by Design of Experiments: the optimized formulation for the codelivery of cannabidiol and paclitaxel (NLC–CBD-PTX), and control formulations, with each active: paclitaxel (NLC-PTX) or cannabidiol (NLC–CBD). These formulations were characterized, and their physicochemical properties are listed in Table 5. The NLC–CBD-PTX formulation had the largest size among all, reaching 206.5 nm, and PDI values did not surpass 0.2, the limit to be considered for a monodisperse population. The ZP values were negative and only approached zero in the case of NLC-PTX (−4.4 mV). Due to the hydrophobicity of the actives, the encapsulation efficiency was very high (≥98%) for both PTX and CBD in the three formulations. Furthermore, coencapsulation did not affect the %EE of the individual actives. Other studies have also demonstrated the colloidal stability and a high encapsulation efficiency of CBD in NLCs, confirming that this DDS is an effective strategy for optimizing the incorporation of highly lipophilic compounds.^64–66^

**Table 5:** Physicochemical Properties (Size, PDI, and ZP) and Encapsulation Efficiency (%EE) of PTX and CBD) in the Formulation Selected by the Factorial Design (NLC–CBD-PTX) and Its Controls (NLC–CBD, NLC-PTX)

formulations	size (nm)	PDI	ZP (mV)	%EE CBD	%EE PTX
NLC–CBD	187.2 ± 1.4	0.139 ± 0.054	–12.8 ± 0.2	98	
NLC-PTX	183.7 ± 4.6	0.171 ± 0.029	–4.4 ± 0.3		99
NLC–CBD-PTX	206.5 ± 1.7	0.193 ± 0.029	–18.2 ± 0.2	98	99

#### 3.2.2 Nanoparticle Tracking Analysis

In the NTA technique, application of the Stokes–Einstein equation allows for the determination of the size (nanometers) of each tracked particle. Furthermore, the specific and individual counting of particles in relation to volume enables a quick determination of nanoparticle concentration in each sample.^51^ By analyzing the distribution of particles based on size (refer to Table 6), one can determine the uniformity of nanoparticle size by the SPAN index that considers the D10, D50, and D90 mean sizes of 10%, 50%, and 90% of the nanoparticles, respectively, accordingly to eq 2.

**Table 6:** NTA Analysis: Mean Size and Size Dispersion at 10% (D10), 50% (D50), and 90% (D90) of the NLC Population, SPAN Index (See Text), and Particle Concentration in the Optimized Formulation and Its Controls (without Paclitaxel or Cannabidiol)

sample	size(nm)	D10(nm)	D50(nm)	D90(nm)	SPAN	concentration (×10^13^ particles/mL)
NLC–CBD	157.2 ± 3.7	112.0 ± 2.8	149.4 ± 4.5	210.6 ± 7.1	0.6	4.60 ± 0.41
NLC-PTX	169.5 ± 2.1	107.2 ± 2.8	169.1 ± 2.5	225.3 ± 2.5	0.7	3.45 ± 0.72
NLC–CBD-PTX	173.4 ± 6.7	89.3 ± 15.9	177.0 ± 3.2	244.0 ± 3.1	0.9	3.44 ± 0.33

The mean diameters determined by NTA were slightly smaller but in good agreement with those obtained from DLS (Figure 2), as expected due to the differing principles of these techniques.^42^ SPAN values below 1 indicate a uniform distribution of particle diameters.^51,67^ Regarding the particle concentration, the formulations showed values on the order of 10^13^ particles/mL (Table 6). These concentrations are consistent with values reported in the literature, for similar NLC systems.^48,51^

#### 3.2.3 X-ray Diffraction

X-ray diffraction analysis (Figure 3) provided information into the crystallinity of the NLC lipid core,^68^ shedding light on the stability of the drug within the particles. Pure myristyl myristate, a key lipid excipient responsible for the solid core of the nanoparticles (28% in the dry mass), exhibited intense peaks at 21 and 24°, confirming its crystalline nature.^45^ However, the intensity of these peaks decreased in the NLC formulations, indicating a reduction in the core’s crystallinity, probably because of the insertion of the actives in between MM, in the core of nanoparticles.^69^ Similarly, the crystalline structure of P68, identified by peaks at 19 and 23°, was also diminished in the NLC formulations; pure PTX displayed weak diffraction peaks at scattering 2θ angles at 5, 7, 10, and 13°,^70^ which were absent in the nanoparticles (NLC-PTX and NLC–CBD-PTX), indicating the partition of PTX into the NLC lipid matrix, considering it is present in a detectable concentration (16% of the NLC dry mass).

**Figure 3:**
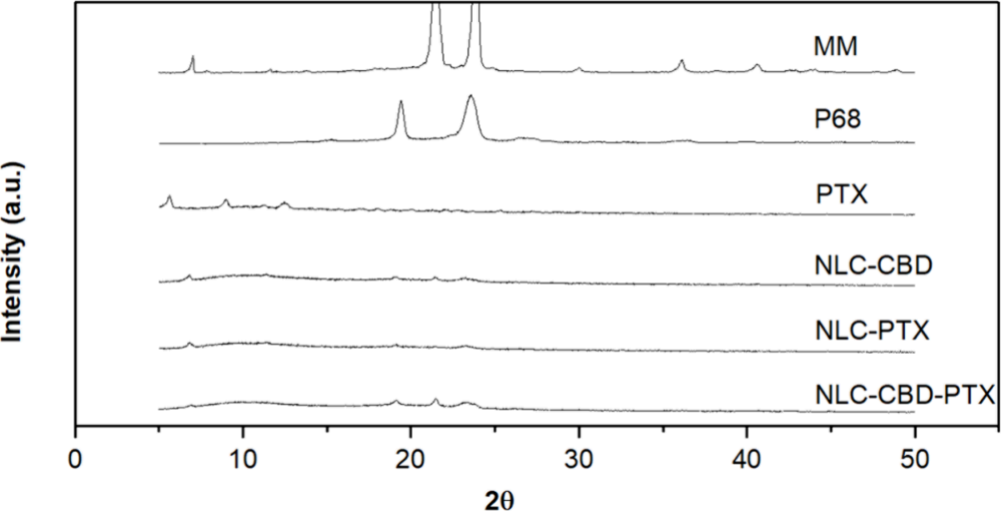
X-ray diffractograms of pure myristyl myristate (MM), pure Pluronic F-68 (P68), pure paclitaxel (PTX), cannabidiol encapsulated in NLCs (NLC–CBD), paclitaxel encapsulated in NLCs (NLC-PTX), and the association of cannabidiol and paclitaxel in NLCs (NLC–CBD-PTX). NLC formulations were freeze-dried before analysis. All diffractograms are in the same scale.

#### 3.2.4 Transmission Electron Microscopy

TEM analyses provided valuable insights into the morphology of NLCs and their nanometric size. Micrographs of diluted NLC formulations with and without PTX are shown in Figure 4, showing predominantly spherical nanoparticles of smooth surfaces, typical of lipid carriers. It is worth noting that the addition of PTX did not change the morphology of the nanoparticles (Figure 4C,D). Their sizes, measured using ImageJ software, were close to ∼200 nm, in good agreement with measurements obtained by DLS and NTA.

**Figure 4:**
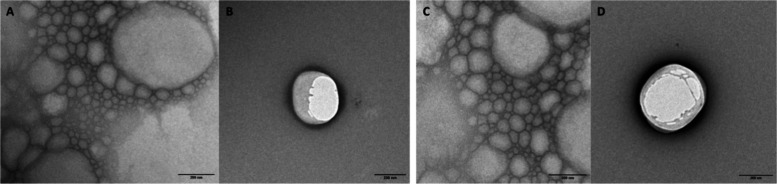
TEM micrographs of NLC formulations without PTX (NLC–CBD, A, B) and with PTX (NLC–CBD-PTX, C, D). Magnifications: 18,500× (A, C) and 23,000× (B, D).

### 3.3 In Vitro Release Experiment

The release profile of drugs encapsulated in NLCs can provide valuable insights into their encapsulation efficiency and release mechanisms.^63^ Figure 5 presents the results obtained for the developed formulation. As shown in Figure 5A, paclitaxel, when formulated in TAXOL, is completely released into the acceptor medium within 24 h of analysis, with an initial rapid release of approximately 60% within the first 6 h of the experiment. As expected, due to the high encapsulation efficiency of PTX in the NLC lipid matrix (99%) and its high lipophilicity, its release from the NLCs occurred more gradually, with only 16% of the drug released after 6 h. Similarly, the results for cannabidiol (Figure 5B) demonstrate that the commercial oily formulation exhibits a prolonged release of 80% in 24 h, whereas when encapsulated in NLCs, only about half of this amount is released within the same period. These findings highlight the ability of NLCs to modulate and sustain the release of lipophilic drugs compared to conventional formulations.^71,72^

**Figure 5:**
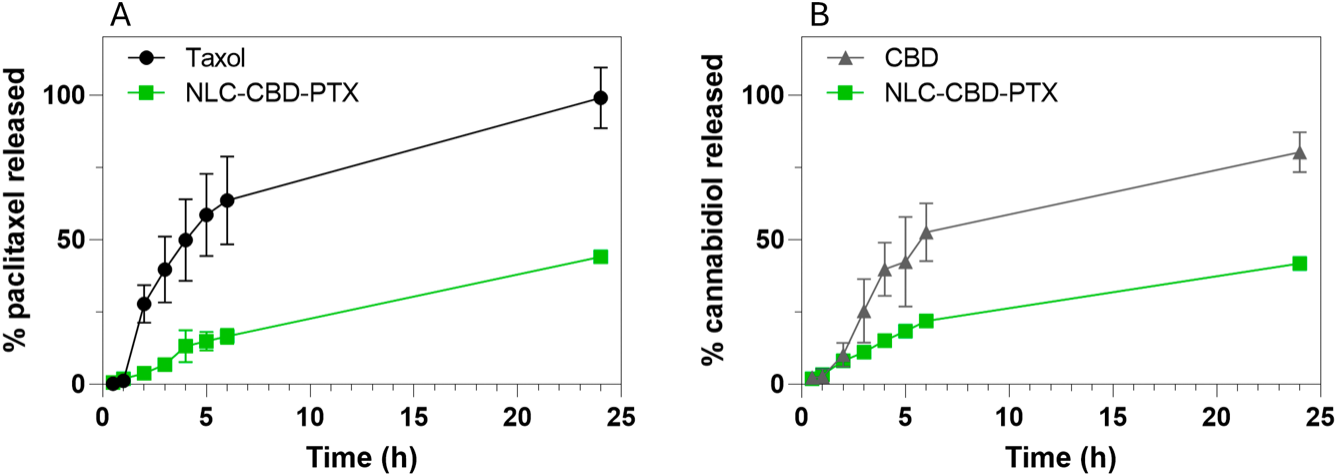
Release profile of paclitaxel (A) and cannabidiol (B) in TAXOL, cannabidiol oil (CBD), and paclitaxel/cannabidiol loaded in NLCs (NLC–CBD-PTX) measured in Franz cells at 37 °C (*n* = 3).

The release curves were analyzed by using various mathematical models with the KinetDS software. The logarithmic model was found to best describe the release profile of the control formulations for both drugs, as indicated by the highest R^2^ values (Table S4). This suggests that the release rate of the control formulations decreases over time. In contrast, the release kinetics of both drugs from the NLC formulations were more appropriately described by the Weibull model. In this model, the shape parameter (β) characterizes the release curve profile. For PTX release, the shape parameter was greater than 1, indicating an initial slow release that accelerates over time, which is consistent with a complex release mechanism such as diffusion through a matrix or erosion. On the other hand, the shape parameter for CBD release was lower than 1, reflecting an initial fast release followed by a slowdown phase.

The results with PTX encapsulated in NLCs are in agreement with other authors ‘reports that NLCs promote a slower release compared to commercial formulations.^73,74^ In the case of cannabidiol, the literature aligns with our findings, showing a slow release of CBD from the NLCs.^65^ More specifically, in the study by Matarazzo et al., an initial rapid release followed by a slower sustained release was observed, consistent with our results.^22^ So, these results are consistent with the encapsulation of drugs in the NLCs’ lipid core but with distinct release behaviors for PTX and CBD, probably influenced by their interactions with the lipid matrix and their physicochemical properties.

### 3.4 Cytotoxicity in the Melanoma Cell Line

To assess the impact of coencapsulation on melanoma cells, the murine melanoma B16–F10 cell line was selected for its widespread use in in vitro testing and its ability to induce tumors in vivo. The cytotoxic effect was measured using the MTT assay,^75,76^ after 24 and 48 h of nanoparticle exposure to the treatments.

Figure 6A, B displays the outcomes of treating B16–F10 cells for 24 and 48 h, respectively, with formulations of free CBD oil and CBD oil encapsulated in NLCs (NLC–CBD). This allowed us to observe the effect of CBD and its encapsulation on this cell line. Burch et al.^77^ demonstrated that CBD oil at a concentration of 0.4 mg/mL inhibits the in vitro growth of B16–F0 melanoma cells. In our study, using a more aggressive melanoma cell line, B16–F10, CBD oil was able to reduce the viability by 50% (IC_50_) at ∼0.9 mg/mL after 24 h of exposure and ∼0.7 mg/mL after 48 h of exposure (Table 7). So, our results align with previous findings in the literature. The encapsulation of CBD in NLCs increased its cytotoxicity (IC_50_ = 0.02 mg/mL after 24 h of exposure and 0.015 mg/mL after 48 h). This finding aligns with the literature, demonstrating that NLCs are capable of enhancing the efficacy of drugs in melanoma cell lines.^78–80^ Furthermore, the control NLC formulation, prepared without CBD but with the same excipient concentration and particle number, exhibited a lower cytotoxic effect compared with NLC–CBD, confirming the specific action of encapsulated CBD on the studied cell line (Figure S4). However, the control NLCs without the drugs were not inert against the studied cell line, which is most probably due to the excipients, as previously reported in the literature.^81^ Nevertheless, the encapsulation of CBD in NLCs significantly enhanced cell death in the studied cell line. Several factors may contribute to this effect, including improved drug bioavailability and enhanced intracellular delivery due to nanoparticle internalization.^82–84^

**Figure 6:**
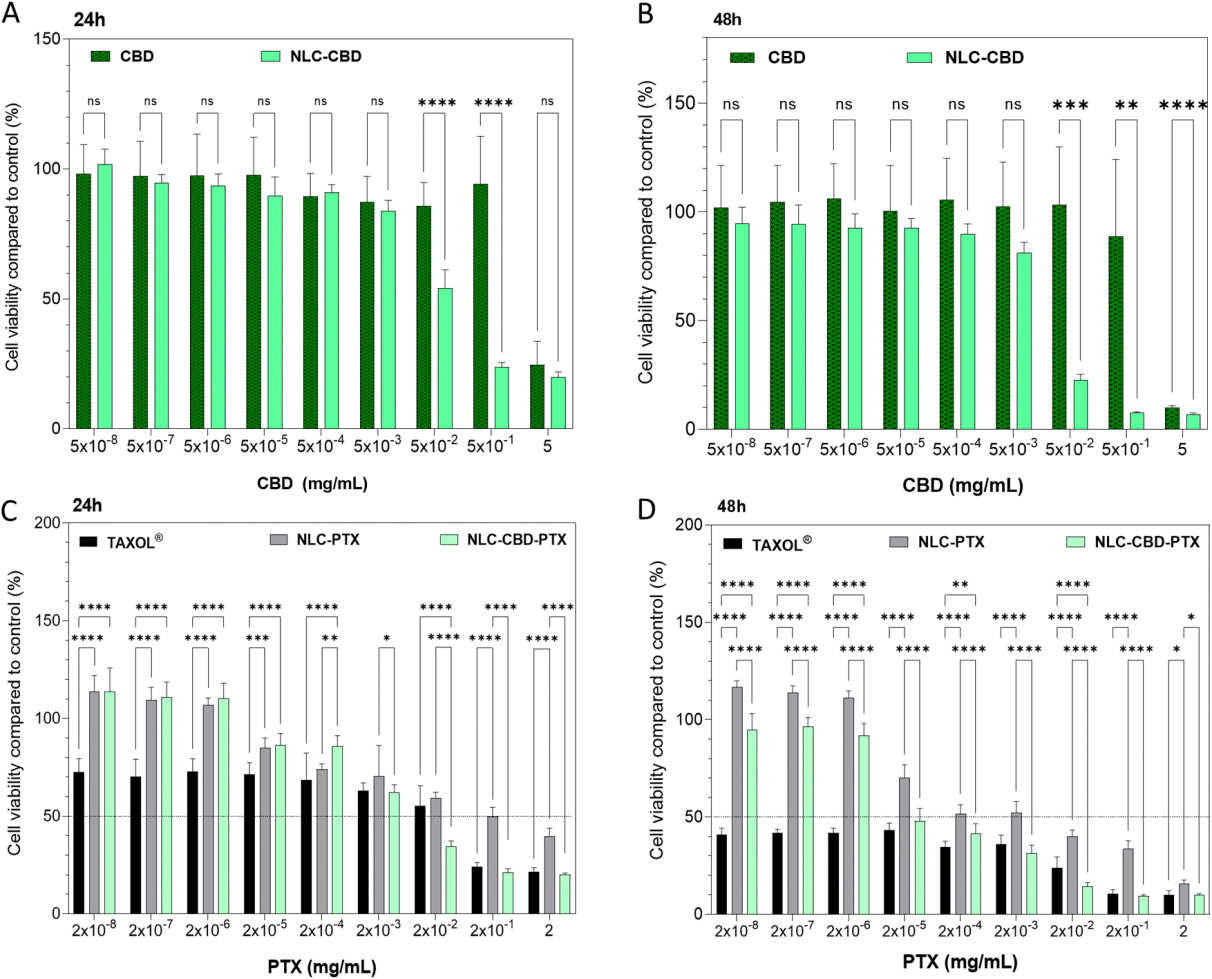
Cell viability (MTT assay) of melanoma strain (B16F10 cells) treated for 24 h (A, C) and 48 h (B, D) with cannabidiol oil (CBD) or encapsulated in NLC (NLC-CDB), paclitaxel commercial (TAXOL) or encapsulated in NLC (NLC-PTX), and the association of cannabidiol and paclitaxel in NLC (NLC–CBD-PTX). Results expressed as mean ± SD (*n* = 12). Statistical analysis by two-way ANOVA plus Tukey-Kramer post hoc. * *p* < 0.05; ** *p* < 0.01; *** *p* < 0.001, **** *p* < 0.0001.

**Table 7:** Half-Maximal Inhibitory Concentration (IC_50_) Values Determined for Cannabidiol Oil Free (CBD) or Encapsulated in NLC (NLC-CDB); Commercial Paclitaxel (TAXOL) or PTX Encapsulated in NLC (NLC-PTX) and the Association of Cannabidiol and Paclitaxel in NLC (NLC–CBD-PTX) against B16F10 Cells, after 24 and 48 h of Treatments, as Measured by the MTT Assay^a^ [Fig fig6]

	IC_50_			
	CBD(mg/mL)		PTX(mg/mL)	
formulation	24 h	48 h	24 h	48 h
CBD	∼0.9	∼0.7		
NLC–CBD	0.02 ± 0.01	0.015 ± 0.003		
TAXOL			3.43 × 10^–3^ ± 6 × 10^–3^	6.86 × 10^–8^ ± 4.28 × 10^–5^
NLC-PTX			0.12 ± 0.14	3.43 × 10^–3^ ± 2.57 × 10^–3^
NLC–CBD-PTX	0.005 ± 0.003	0.0003 ± 0.0001	6.86 × 10^–3^ ± 3.43 × 10^–3^	0.86 × 10^–4^ ± 4.28 × 10^–5^

a Analyses were performed with GraphPad Prism 9.3.0 software with values of the curves in Figure 6.

The formulations containing paclitaxel in the commercial formulation, TAXOL, and encapsulated (NLC-PTX) were tested after 24 and 48 h of exposure (Figure 6C,D, respectively). The reference drug TAXOL is a micellar formulation of Cremophor EL (polyoxyethylated castor oil) in an ethanol:water solution, which evidently causes intense cell death even at low concentrations: IC_50_ of 3.43 × 10^–3^ and 6.86 × 10^–8^ mg/mL at 24 and 48 h, respectively. In this commercial formulation, in addition to a high concentration of Cremophor EL, which forms micelles, ethanol is also present, potentially increasing the toxic effects of the drug.^85,86^ PTX encapsulated in NLCs mitigates the drastic cell death seen in the TAXOL formulation, probably due to its less toxic excipients, showing IC_50_ values of 1.2 × 10^–1^ and 3.43 × 10^–3^ mg/mL after 24 and 48 h of exposure, respectively. In this context, the literature highlights the advantages of encapsulating PTX in NLCs over the commercial formulation, mainly due to reduced toxicity, with reports indicating lower systemic toxicity as well.^37^

The optimized formulation (NLC–CBD-PTX) demonstrated higher cytotoxicity compared to commercial formulations and formulations of NLCs with the active ingredients (NLC–CBD and NLC-PTX) separated. There was a 75% decrease in the IC_50_ of CBD (0.005 mg/mL) after 24 h of exposure and a 98% decrease (0.0002 mg/mL) after 48 h of exposure. As for PTX, the decrease in IC_50_ was of 94 and 98% after 24 and 48 h of exposure, respectively. In other words, a lower concentration of both active ingredients is required to cause 50% of cell death when they are coencapsulated in the same formulation. Therefore, these data demonstrate a greater efficacy in reducing cell viability for NLC–CBD-PTX compared to the drugs in their commercial formulations.

Although the final phenotype of cell viability indicates superior efficacy of the NLC–CBD-PTX formulation compared to the commercial (TAXOL and CBD) controls in the tested cell line, the combination index (CI) was assessed to distinguish the mechanisms of drug interactions (CI < 1, synergy; CI = 1, additivity; and CI > 1, antagonism).^87^ After 24 h of testing, the CI at the three highest concentrations of NLC–CBD-PTX was found to be <1 (Figure S5). Similarly, after 48 h, the CI at the two highest concentrations of NLC–CBD-PTX remained below 1. Thus, at the higher concentrations tested, where greater efficacy in terms of reduced cell viability was observed, the CI values suggest synergy between the drugs encapsulated in NLCs compared to the commercial formulations.

In the comparison of the drug combination in NLC–CBD-PTX with their respective controls in NLC (NLC–CBD and NLC-PTX) after 24 h of exposure, the CI results mirrored the behavior observed when compared to the commercial formulations, with CI < 1 at the highest concentrations (Figure S6). However, after 48 h of exposure, this effect was no longer observed. Instead, an antagonistic effect emerged at these concentrations, with CI > 1. This suggests that prolonged exposure times influence the drug combination differently than when the drugs are individually encapsulated in NLCs. As this is the first study to investigate such a drug combination in NLCs, these findings are particularly significant and warrant further exploration into the mechanisms underlying this response, particularly with respect to how exposure time modulates the combinatory effects within the nanometric system

These results demonstrate the successful development and optimization of NLCs coencapsulating CBD and PTX, two highly hydrophobic active compounds. The formulation exhibited good physicochemical properties, including optimal particle size, polydispersity index (PDI), and ζ potential (ZP), ensuring stability over 60 days. The encapsulation efficiency of both drugs was exceptionally high (≥98%), and the NLC system effectively modulated the release of PTX and CBD, providing a sustained release profile compared to their commercial counterparts. Notably, the NLC–CBD-PTX formulation exhibited enhanced cytotoxicity against the B16–F10 melanoma cell line, with significantly lower IC_50_ values compared to both the individual NLC formulations (NLC–CBD and NLC-PTX) and the commercial formulations. This suggests a synergistic effect (CI < 1) between CBD and PTX when coencapsulated, particularly at higher concentrations and shorter exposure times. However, the combination index revealed that prolonged exposure (48 h) of the cells led to an antagonistic effect relatively to the individual (NLC–CBD and NLC-PTX) formulations, highlighting the importance of more studies about the exposure time in drug interactions in this DDS.

Although this study represents an initial investigation into the combination of a chemotherapeutic agent and a cannabinoid compound in NLCs, it opens promising avenues for further mechanistic studies to elucidate the underlying factors contributing to the observed synergy and antagonism between CBD and PTX within the NLC system. More specific investigations, utilizing a broader range of dose combinations and comparisons with the free drugs (rather than commercial formulations, as in this study), should be pursued in future work. Overall, this study establishes a robust foundation for the development of advanced nanomedicines for cancer therapy, offering significant prospects for future research and clinical translation, particularly in the context of cannabinoid-based combinations.

## 4 Conclusions

Based on the obtained results, nanostructured lipid carriers coencapsulating paclitaxel and cannabidiol were successfully developed and characterized, aiming to enhance the anticancer properties and reduce the side effects associated with chemotherapy. Physicochemical analyses conducted using techniques such as DLS, NTA, TEM, and XRD demonstrated the feasibility of the developed system. Cytotoxicity assays confirmed the antitumor efficacy and synergistic action of the coencapsulated actives at high doses and shorter exposure times (24 h), highlighting the potential of this system for future in vivo testing, particularly in chemo-resistant tumors. Additionally, the addition of CBD to conventional (PTX) treatment in a single formulation not only offers benefits in chemotherapy due to the need for a single administration but also presents a promising strategy to reduce PTX-associated side effects such as peripheral neuropathy. Furthermore, this study demonstrates the therapeutic potential of cannabidiol when combined with a classic antineoplastic, offering not only a pharmaceutical option just in palliative care situations but also the ability to act in earlier stages of chemotherapy treatment. So, these advancements contribute to the development of more effective and less aggressive treatments for oncology patients, underscoring the potential of NLCs as therapeutic vehicles.

## Supplementary Material



## Notes

Chromatogram of PTX and CBD (Figure S1), predicted vs actual results (Figure S2), desirability graph (Figure S3), cell viability graph (Figure S4), drug combination results in NLC–CBD-PTX compared with commercial formulations (Figure S5), drug combination results in NLC–CBD-PTX compared with NLC controls (Figure S6), factorial model tables for size (Table S1), PDI (Table S2) and ZP (Table S3), and *R* ^2^ coefficients of mathematical models applied to the in vitro release kinetic curves (Table S4) (PDF)

## References

[1] Pereira I., Monteiro C., Pereira-Silva M., Peixoto D., Nunes C., Reis S., Veiga F., Hamblin M. R., Paiva-Santos A. C. (2023). Nanodelivery
Systems for Cutaneous Melanoma Treatment. Eur.
J. Pharm. Biopharm..

[2] International Agency for Research on Cancer; World Health Organization. Skin Cancer. https://www.iarc.who.int/cancer-type/skin-cancer/ (accessed 2024–11–12).

[3] Melanoma - Guidelines for Patients Details, https://www.nccn.org/patientresources/patient-resources/guidelines-for-patients/guidelines-for-patients-details?patientGuidelineId=21 (accessed January 21, 2025).

[4] Cutaneous Melanome, https://www.nccn.org/professionals/physician_gls/pdf/cutaneous_melanoma.pdf.

[5] Alves R. C., Fernandes R. P., Eloy J. O., Salgado H. R. N., Chorilli M. (2018). Characteristics,
Properties and Analytical Methods of Paclitaxel: A Review. Crit Rev. Anal Chem..

[6] Khanna C., Rosenberg M., Vail D. M. (2015). A Review of Paclitaxel and Novel
Formulations Including Those Suitable for Use in Dogs. J. Vet Intern Med..

[7] Alqahtani, F. Y. ; Aleanizy, F. S. ; El Tahir, E. ; Alkahtani, H. M. ; AlQuadeib, B. T. Paclitaxel. In Profiles of Drug Substances, Excipients and Related Methodology; Elsevier, 2019; Vol. 44, pp 205–238. 10.1016/bs.podrm.2018.11.001. 31029218

[8] Yu D.-L., Lou Z.-P., Ma F.-Y., Najafi M. (2022). The Interactions of
Paclitaxel with Tumour Microenvironment. Int.
Immunopharmacol.

[9] Singla A. K., Garg A., Aggarwal D. (2002). Paclitaxel and Its
Formulations. Int. J. Pharm..

[10] Ghadi R., Dand N. (2017). BCS Class IV Drugs:
Highly Notorious Candidates for Formulation Development. J. Controlled Release.

[11] Bhalani D. V., Nutan B., Kumar A., Singh Chandel A. K. (2022). Bioavailability
Enhancement Techniques for Poorly Aqueous Soluble Drugs and Therapeutics. Biomedicines.

[12] Panchagnula R. (1998). Pharmaceutical
Aspects of Paclitaxel. Int. J. Pharm..

[13] Surapaneni M. S., Das S. K., Das N. G. (2012). Designing Paclitaxel
Drug Delivery
Systems Aimed at Improved Patient Outcomes: Current Status and Challenges. ISRN Pharmacol.

[14] Marzo I., Naval J. (2013). Antimitotic Drugs in Cancer Chemotherapy: Promises and Pitfalls. Biochem. Pharmacol..

[15] Costa A. M., Silva V. V. (2017). Estratégias
Nanotecnológicas Para Diagnóstico
E Tratamento Do Câncer. Revista Saúde
e Meio Ambiente.

[16] Valenti, C. ; Billi, M. ; Pancrazi, G. L. ; Calabria, E. ; Armogida, N. G. ; Tortora, G. ; Pagano, S. ; Barnaba, P. ; Marinucci, L. Biological Effects of Cannabidiol on Human Cancer Cells: Systematic Review of the Literature. Pharmacol. Res. 2022, 181. 106267 10.1016/j.phrs.2022.106267. 35643249

[17] Morales P., Jagerovic N. (2020). Novel Approaches and Current Challenges
with Targeting
the Endocannabinoid System. Expert Opin Drug
Discov.

[18] Barrie N., Manolios N. (2017). The Endocannabinoid System in Pain
and Inflammation:
Its Relevance to Rheumatic Disease. Eur. J.
Rheumatol.

[19] Basavarajappa B. S., Shivakumar M., Joshi V., Subbanna S. (2017). Endocannabinoid System
in Neurodegenerative Disorders. J. Neurochem.

[20] Fraguas-Sánchez A. I., Martín-Sabroso C., Torres-Suárez A. I. (2018). Insights
into the Effects of the Endocannabinoid System in Cancer: A Review. Br. J. Pharmacol..

[21] Coelho M. P., Duarte P., Calado M., Almeida A. J., Reis C. P., Gaspar M. M. (2023). The Current Role
of Cannabis and Cannabinoids in Health:
A Comprehensive Review of Their Therapeutic Potential. Life Sci..

[22] Matarazzo A. P., Elisei L. M. S., Carvalho F. C., Bonfílio R., Ruela A. L. M., Galdino G., Pereira G. R. (2021). Mucoadhesive Nanostructured
Lipid Carriers as a Cannabidiol Nasal Delivery System for the Treatment
of Neuropathic Pain. European Journal of Pharmaceutical
Sciences.

[23] Hasan N., Imran M., Sheikh A., Tiwari N., Jaimini A., Kesharwani P., Jain G. K., Ahmad F. J. (2023). Advanced Multifunctional
Nano-Lipid Carrier Loaded Gel for Targeted Delivery of 5-Flurouracil
and Cannabidiol against Non-Melanoma Skin Cancer. Environ. Res..

[24] Galeano M., Vaccaro F., Irrera N., Caradonna E., Borgia F., Li Pomi F., Squadrito F., Vaccaro M. (2024). Melanoma and Cannabinoids: A Possible Chance for Cancer
Treatment. Exp Dermatol.

[25] Mangal N., Erridge S., Habib N., Sadanandam A., Reebye V., Sodergren M. H. (2021). Cannabinoids
in the Landscape of
Cancer. J. Cancer Res. Clin Oncol.

[26] Millar S. A., Stone N. L., Bellman Z. D., Yates A. S., England T. J., O’Sullivan S. E. (2019). A Systematic Review of Cannabidiol Dosing in Clinical
Populations. Br. J. Clin. Pharmacol..

[27] Koch N., Jennotte O., Gasparrini Y., Vandenbroucke F., Lechanteur A., Evrard B. (2020). Cannabidiol Aqueous
Solubility Enhancement:
Comparison of Three Amorphous Formulations Strategies Using Different
Type of Polymers. Int. J. Pharm..

[28] Rebelatto E. R. L., Rauber G. S., Caon T. (2023). An Update
of Nano-Based Drug Delivery
Systems for Cannabinoids: Biopharmaceutical Aspects & Therapeutic
Applications. Int. J. Pharm..

[29] Millar S. A., Maguire R. F., Yates A. S., O’sullivan S. E. (2020). Towards
Better Delivery of Cannabidiol (Cbd). Pharmaceuticals.

[30] Eloy, J. d. O. Lipossomas e Imunolipossomas Contendo Fármacos Antitumorais: Desenvolvimento, Caracterização e Avaliação Da Eficácia Contra o Câncer de Mama Mama; FCF - Universidade de São Paulo - USP, 2016.

[31] Izza N., Watanabe N., Okamoto Y., Suga K., Wibisono Y., Kajimura N., Mitsuoka K., Umakoshi H. (2022). Dependence of the Core–Shell
Structure on the Lipid Composition of Nanostructured Lipid Carriers:
Implications for Drug Carrier Design. ACS Appl.
Nano Mater..

[32] Tagde P., Najda A., Nagpal K., Kulkarni G. T., Shah M., Ullah O., Balant S., Rahman M. H. (2022). Nanomedicine-Based
Delivery Strategies for Breast Cancer Treatment and Management. Int. J. Mol. Sci..

[33] Kitamura T., Qian B. Z., Pollard J. W. (2015). Immune
Cell Promotion of Metastasis. Nat. Rev. Immunol.

[34] Hanahan D., Weinberg R. A. (2011). Hallmarks of Cancer:
The next Generation. Cell.

[35] Khosa A., Reddi S., Saha R. N. (2018). Nanostructured
Lipid Carriers for
Site-Specific Drug Delivery. Biomedicine &
Pharmacotherapy.

[36] Cho K., Wang X., Nie S., Chen Z., Shin D. M. (2008). Therapeutic
Nanoparticles for Drug Delivery in Cancer. Clin.
Cancer Res..

[37] Rodrigues
da Silva G. H., de Moura L. D., de Carvalho F. V., Geronimo G., Mendonça T. C., de Lima F. F., de Paula E. (2021). Antineoplastics
Encapsulated in Nanostructured Lipid Carriers. Molecules.

[38] Xu M., Li G., Zhang H., Chen X., Li Y., Yao Q., Xie M. (2020). Sequential
Delivery of Dual Drugs with Nanostructured Lipid Carriers
for Improving Synergistic Tumor Treatment Effect. Drug Delivery.

[39] Meng T., Li J., Qi X. (2014). Preparation
and Evaluation of Lipid-Matrix Nanocarrier
Co-Delivery Gene and Sensibilizer to Elevate Docetaxel Antitumor. J. Chin. Pharm. Sci..

[40] Marzęda P., Drozd M., Wróblewska-Łuczka P., Łuszczki J. J. (2021). Cannabinoids and Their Derivatives in Struggle against
Melanoma. Pharmacological Reports.

[41] Schwarz C., Mehnert W., Lucks J. S., Müller R. H. (1994). Solid Lipid
Nanoparticles (SLN) for Controlled Drug Delivery. I. Production, Characterization
and Sterilization. J. Controlled Release.

[42] Sharma A., Baldi A. (2018). Nanostructured Lipid Carriers: A Review. J.
Dev. Drugs.

[43] Javed S., Mangla B., Almoshari Y., Sultan M. H., Ahsan W. (2022). Nanostructured
Lipid Carrier System: A Compendium of Their Formulation Development
Approaches, Optimization Strategies by Quality by Design, and Recent
Applications in Drug Delivery. Nanotechnol Rev..

[44] de
Moura L. D., Ribeiro L. N. M., de Carvalho F. V., Rodrigues da Silva G. H., Lima Fernandes P. C., Brunetto S. Q., Ramos C. D., Velloso L. A., de Araújo D. R., de Paula E. (2021). Docetaxel and Lidocaine Co-Loaded (Nlc-in-Hydrogel)
Hybrid System Designed for the Treatment of Melanoma. Pharmaceutics.

[45] de
Carvalho F. V., Ribeiro L. N. d. M., de Moura L. D., Rodrigues
da Silva G. H., Mitsutake H., Mendonça T. C., Geronimo G., Breitkreitz M. C., de Paula E. (2022). Docetaxel Loaded in
Copaiba Oil-Nanostructured Lipid Carriers as a Promising DDS for Breast
Cancer Treatment. Molecules.

[46] Fukuda I. M., Pinto C. F. F., Moreira C. D. S., Saviano A. M., Lourenço F. R. (2018). Design
of Experiments (DoE) Applied to Pharmaceutical and Analytical Quality
by Design (QbD). Braz. J. Pharm. Sci..

[47] Tavares
Luiz M., Santos Rosa Viegas J., Palma Abriata J., Viegas F., Testa Moura de Carvalho Vicentini F., Lopes Badra Bentley M. V., Chorilli M., Maldonado
Marchetti J., Tapia-Blácido D. R. (2021). Design of Experiments
(DoE) to Develop and to Optimize Nanoparticles as Drug Delivery Systems. Eur. J. Pharm. Biopharm..

[48] Rodrigues
da Silva G. H., Ribeiro L. N. M., Mitsutake H., Guilherme V. A., Castro S. R., Poppi R. J., Breitkreitz M. C., de Paula E., Optimised N. L. C. (2017). A Nanotechnological Approach to Improve
the Anaesthetic Effect of Bupivacaine. Int.
J. Pharm..

[49] Müller R. H., Mäder K., Gohla S. (2000). Solid Lipid Nanoparticles (SLN) for
Controlled Drug Delivery–a Review of the State of the Art. Eur. J. Pharm. Biopharm..

[50] Rodrigues
da Silva G. H., Geronimo G., García-López J. P., Ribeiro L. N. M., de Moura L. D., Breitkreitz M. C., Feijóo C. G., de Paula E. (2020). Articaine in Functional NLC Show
Improved Anesthesia and Anti-Inflammatory Activity in Zebrafish. Sci. Rep.

[51] Ribeiro L. N. D. M., Couto V. M., Fraceto L. F., De Paula E. (2018). Use of Nanoparticle
Concentration as a Tool to Understand the Structural Properties of
Colloids. Sci. Rep.

[52] Li M., Wilkinson D., Patchigolla K. (2005). Comparison of Particle Size Distributions
Measured Using Different Techniques. Particulate
Science and Technology.

[53] Schneider C. A., Rasband W. S., Eliceiri K. W. (2012). NIH Image
to ImageJ: 25 Years of
Image Analysis. Nat. Methods.

[54] Makeen H. A., Mohan S., Al-Kasim M. A., Attafi I. M., Ahmed R. A., Syed N. K., Sultan M. H., Al-Bratty M., Alhazmi H. A., Safhi M. M., Ali R., Intakhab
Alam M. (2020). Gefitinib Loaded Nanostructured Lipid Carriers: Characterization,
Evaluation and Anti-Human Colon Cancer Activity in Vitro. Drug Deliv.

[55] Rawal S., Patel B., Patel M. M. (2020). Fabrication, Optimisation
and in
Vitro Evaluation of Docetaxel and Curcumin Co-Loaded Nanostructured
Lipid Carriers for Improved Antitumor Activity against Non-Small Cell
Lung Carcinoma. J. Microencapsul.

[56] Lv W., Zhao S., Yu H., Li N., Garamus V. M., Chen Y., Yin P., Zhang R., Gong Y., Zou A. (2016). Brucea Javanica Oil-Loaded Nanostructure
Lipid Carriers (BJO NLCs):
Preparation, Characterization and in Vitro Evaluation. Colloids Surf. A Physicochem Eng. Asp.

[57] Mendyk A., Jachowicz R., Fijorek K., Dorozyński P., Kulinowski P., Polak S. (2012). KinetDS: An Open Source Software
for Dissolution Test Data Analysis. Dissolut
Technol..

[58] Jaber N., Aiedeh K. (2019). Sorption Behavior and Release Kinetics of Iron (II)
Ions by Oleoyl Chitosan Polymeric Nanoparticles. J. Drug Deliv Sci. Technol..

[59] Mosmann T. (1983). Rapid Colorimetric
Assay for Cellular Growth and Survival: Application to Proliferation
and Cytotoxicity Assays. J. Immunol Methods.

[60] Spinozzi G., Tini V., Ferrari A., Gionfriddo I., Ranieri R., Milano F., Pierangeli S., Donnini S., Mezzasoma F., Silvestri S., Falini B., Martelli M. P. (2022). SiCoDEA: A Simple, Fast and Complete
App for Analyzing the Effect of Individual Drugs and Their Combinations. Biomolecules.

[61] Han F., Li S., Yin R., Liu H., Xu L. (2008). Effect of Surfactants
on the Formation and Characterization of a New Type of Colloidal Drug
Delivery System: Nanostructured Lipid Carriers. Colloids Surf. A Physicochem Eng. Asp.

[62] de
Castro K. C., Coco J. C., dos Santos É. M., Ataide J. A., Martinez R. M., do Nascimento M. H. M., Prata J., da Fonte P. R. M. L., Severino P., Mazzola P. G., Baby A. R., Souto E. B., de Araujo D. R., Lopes A. M. (2023). Pluronic® Triblock Copolymer-Based Nanoformulations
for Cancer Therapy: A 10-Year Overview. J. Controlled
Release.

[63] Rodrigues
da Silva G. H., Lemes J. B. P., Geronimo G., Freitas
de Lima F., de Moura L. D., Carvalho dos Santos A., Carvalho N. S., Malange K. F., Breitkreitz M. C., Parada C. A., de Paula E. (2021). Lipid Nanoparticles Loaded with Butamben
and Designed to Improve Anesthesia at Inflamed Tissues. Biomater Sci..

[64] Grifoni L., Vanti G., Bilia A. R. (2023). Nanostructured
Lipid Carriers Loaded
with Cannabidiol Enhance Its Bioaccessibility to the Small Intestine. Nutraceuticals.

[65] Morakul B., Junyaprasert V. B., Sakchaisri K., Teeranachaideekul V. (2023). Cannabidiol-Loaded
Nanostructured Lipid Carriers (NLCs) for Dermal Delivery: Enhancement
of Photostability, Cell Viability, and Anti-Inflammatory Activity. Pharmaceutics.

[66] Taha I. E., ElSohly M. A., Radwan M. M., Elkanayati R. M., Wanas A., Joshi P. H., Ashour E. A. (2024). Enhancement of Cannabidiol
Oral Bioavailability through the Development of Nanostructured Lipid
Carriers: In Vitro and in Vivo Evaluation Studies. Drug Deliv Transl Res..

[67] Bender E. A., Adorne M. D., Colomé L. M., Abdalla D. S. P., Guterres S. S., Pohlmann A. R. (2012). Hemocompatibility
of Poly­(ε-Caprolactone) Lipid-Core
Nanocapsules Stabilized with Polysorbate 80-Lecithin and Uncoated
or Coated with Chitosan. Int. J. Pharm..

[68] Bunjes H. (2011). Structural
Properties of Solid Lipid Based Colloidal Drug Delivery Systems. Curr. Opin. Colloid Interface Sci..

[69] Muller R. H., Shegokar R., Keck C. M. (2011). 20 Years
of Lipid Nanoparticles (SLN
& NLC): Present State of Development & Industrial Applications. Curr. Drug Discovery Technol..

[70] Bang K. H., Na Y. G., Huh H. W., Hwang S. J., Kim M. S., Kim M., Lee H. K., Cho C. W. (2019). The Delivery
Strategy of Paclitaxel
Nanostructured Lipid Carrier Coated with Platelet Membrane. Cancers.

[71] Souto E. B., Wissing S. A., Barbosa C. M., Müller R. H. (2004). Development
of a Controlled Release Formulation Based on SLN and NLC for Topical
Clotrimazole Delivery. Int. J. Pharm..

[72] Rapalli V. K., Kaul V., Waghule T., Gorantla S., Sharma S., Roy A., Dubey S. K., Singhvi G. (2020). Curcumin Loaded Nanostructured Lipid
Carriers for Enhanced Skin Retained Topical Delivery: Optimization,
Scale-up, in-Vitro Characterization and Assessment of Ex-Vivo Skin
Deposition. European Journal of Pharmaceutical
Sciences.

[73] Yang X. Y., Li Y. X., Li M., Zhang L., Feng L. X., Zhang N. (2013). Hyaluronic Acid-Coated Nanostructured Lipid Carriers for Targeting
Paclitaxel to Cancer. Cancer Lett..

[74] Marathe S., Shadambikar G., Mehraj T., Sulochana S. P., Dudhipala N., Majumdar S. (2022). Development of α-Tocopherol
Succinate-Based Nanostructured Lipid Carriers for Delivery of Paclitaxel. Pharmaceutics.

[75] Doktorovova S., Souto E. B., Silva A. M. (2014). Nanotoxicology Applied
to Solid Lipid
Nanoparticles and Nanostructured Lipid Carriers - A Systematic Review
of in Vitro Data. Eur. J. Pharm. Biopharm..

[76] Sarma A., Bania R., Devi J. R., Deka S. (2021). Therapeutic Nanostructures
and Nanotoxicity. Journal of Applied Toxicology.

[77] Burch R., Mortuza A., Blumenthal E., Mustafa A. (2021). Effects of Cannabidiol
(CBD) on the Inhibition of Melanoma Cells in Vitro. J. Immunoassay Immunochem.

[78] Mohammadian J., Mahmoudi S., Pourmohammad P., Pirouzpanah M., Salehnia F., Maroufi N. F., Samadi N., Sabzichi M. (2020). Formulation
of Stattic as STAT3 Inhibitor in Nanostructured Lipid Carriers (NLCs)
Enhances Efficacy of Doxorubicin in Melanoma Cancer Cells. Naunyn Schmiedebergs Arch Pharmacol.

[79] Malta R., Loureiro J. B., Costa P., Sousa E., Pinto M., Saraiva L., Amaral M. H. (2021). Development
of Lipid Nanoparticles
Containing the Xanthone LEM2 for Topical Treatment of Melanoma. J. Drug Deliv Sci. Technol..

[80] Cocoş F.-I., Anuţa V., Popa L., Ghica M. V., Nica M.-A., Mihăilă M., Fierăscu R. C., Trică B., Nicolae C. A., Dinu-Pîrvu C.-E. (2024). Development
and Evaluation of Docetaxel-Loaded Nanostructured Lipid Carriers for
Skin Cancer Therapy. Pharmaceutics.

[81] Rathee J., Kanwar R., Kumari L., Pawar S. V., Sharma S., Ali Md. E., Salunke D. B., Mehta S. K. (2023). Development of Nanostructured
Lipid Carriers as a Promising Tool for Methotrexate Delivery: Physicochemical
and in Vitro Evaluation. J. Biomol Struct Dyn.

[82] Deng C., Jia M., Wei G., Tan T., Fu Y., Gao H., Sun X., Zhang Q., Gong T., Zhang Z. (2017). Inducing Optimal Antitumor
Immune Response through Coadministering IRGD with Pirarubicin Loaded
Nanostructured Lipid Carriers for Breast Cancer Therapy. Mol. Pharmaceutics.

[83] Garbuzenko O. B., Kuzmov A., Taratula O., Pine S. R., Minko T. (2019). Strategy to
Enhance Lung Cancer Treatment by Five Essential Elements: Inhalation
Delivery, Nanotechnology, Tumor-Receptor Targeting, Chemo- and Gene
Therapy. Theranostics.

[84] Chaudhari V. S., Gawali B., Saha P., Naidu V. G. M., Murty U. S., Banerjee S. (2021). Quercetin and Piperine
Enriched Nanostructured Lipid
Carriers (NLCs) to Improve Apoptosis in Oral Squamous Cellular Carcinoma
(FaDu Cells) with Improved Biodistribution Profile. Eur. J. Pharmacol..

[85] Sharma A., Straubinger R. M. (1994). Novel Taxol Formulations: Preparation
and Characterization
of Taxol-Containing Liposomes. Pharm. Res..

[86] Nygren P., Csoka K., Jonsson B., Fridborg H., Bergh J., Hagberg H., Glimelius B., Brodin O., Tholander B., Kreuger A., Lönnerholm G., Jakobsson A., Olsen L., Kristensen J., Larsson R. (1995). The Cytotoxic Activity
of Taxol in Primary Cultures of Tumour Cells from Patients Is Partly
Mediated by Cremophor EL. Br. J. Cancer.

[87] Chou T.-C. (2010). Drug Combination
Studies and Their Synergy Quantification Using the Chou-Talalay Method. Cancer Res..

