# Supplementary material for: Codelivery of Paclitaxel and Cannabidiol in Lipid Nanoparticles Enhances Cytotoxicity against Melanoma Cells
Source: ACS Omega. 2025 May 22;10(21):21568–80. doi: 10.1021/acsomega.5c00689 (PMC12138705; doi:10.1021/acsomega.5c00689)
Supplement: Supplementary file 1 [file ao5c00689_si_007.pdf]

SUPPLEMENTARY MATERIALS

Codelivery of Paclitaxel and Cannabidiol in Lipid Nanoparticles Enhances Cytotoxicity Against Melanoma Cells

Fabíola V. de Carvalho[a†], Gabriela Geronimo[a†], Ludmilla D. de Moura[a], Talita C. Mendonça[a], Márcia Cristina Breitkreitz[c], Eneida de Paula[a*] & Gustavo H. Rodrigues da Silva[a,b*]

[a] Department of Biochemistry and Tissue Biology, Institute of Biology, University of Campinas (UNICAMP), 13083-862 Campinas-SP, Brazil.

[b] Brazilian Biosciences National Laboratory, Brazilian Center for Research in Energy and Materials, 13083-100 Campinas-SP, Brazil.

[c] Department of Analytical Chemistry, Institute of Chemistry, University of Campinas (UNICAMP), 13083-970 Campinas-SP, Brazil.

[†] These authors contributed equally.

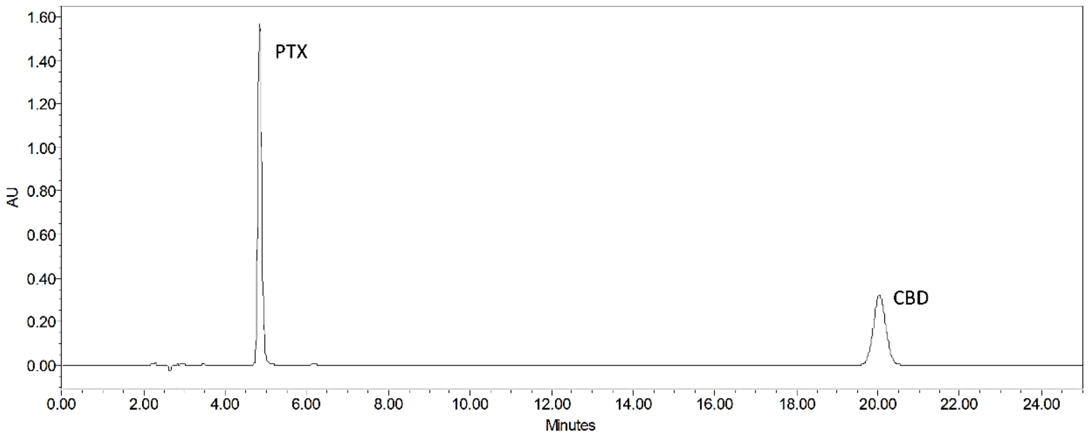

Figure S1 - Chromatogram showing the separation of paclitaxel (PTX) and cannabidiol (CBD) peaks at 4.9 and 20 min, respectively, in the NLC-CBD-PTX sample.

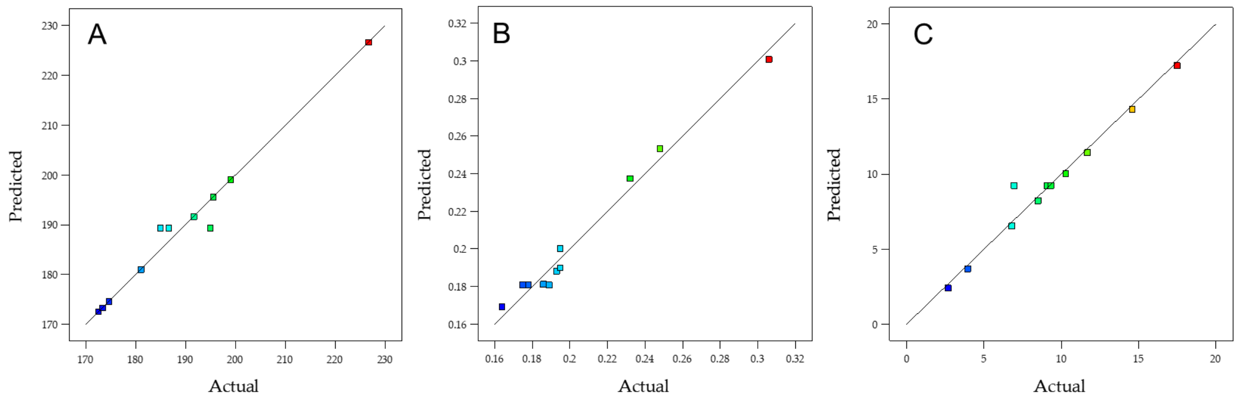

Figure S2 - Predicted vs actual results for Particle size (A); PDI (B) and Zeta potential (C).

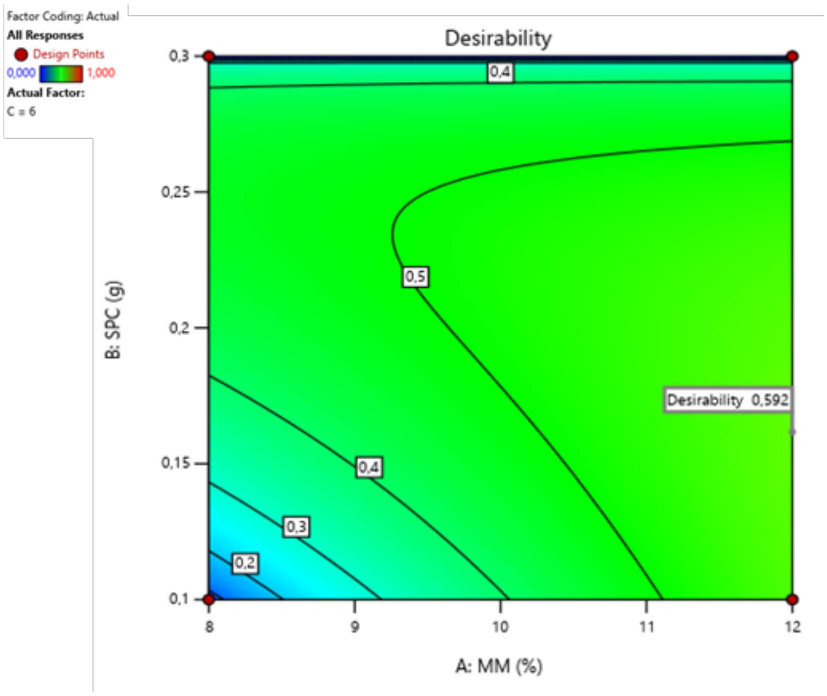

Figure S3 - Desirability graph of the NLC-CBD-PTX factorial design. P68 concentration was kept in 6 %.

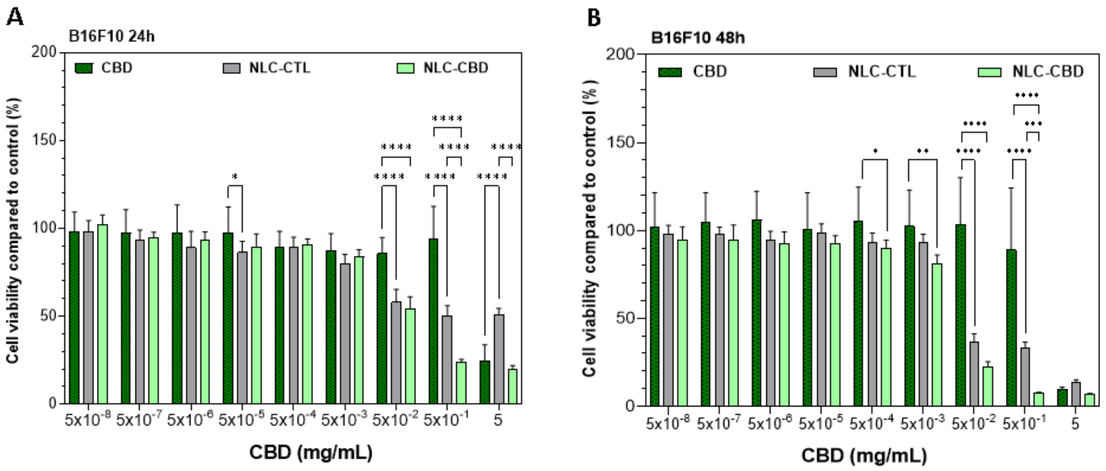

Figure S4 - Cell viability (MTT assay) of melanoma strain (B16F10 cells) treated for 24 h (**A**) and 48 h (**B**) with control samples: CBD, NLC-CTL and NLC-CBD. Results expressed as mean ± SD (n = 12). Statistical analysis by Two-way ANOVA plus Tukey-Kramer post hoc. * $p < 0.05$; ** $p < 0.01$; *** $p < 0.001$. **** $p < 0.0001$.

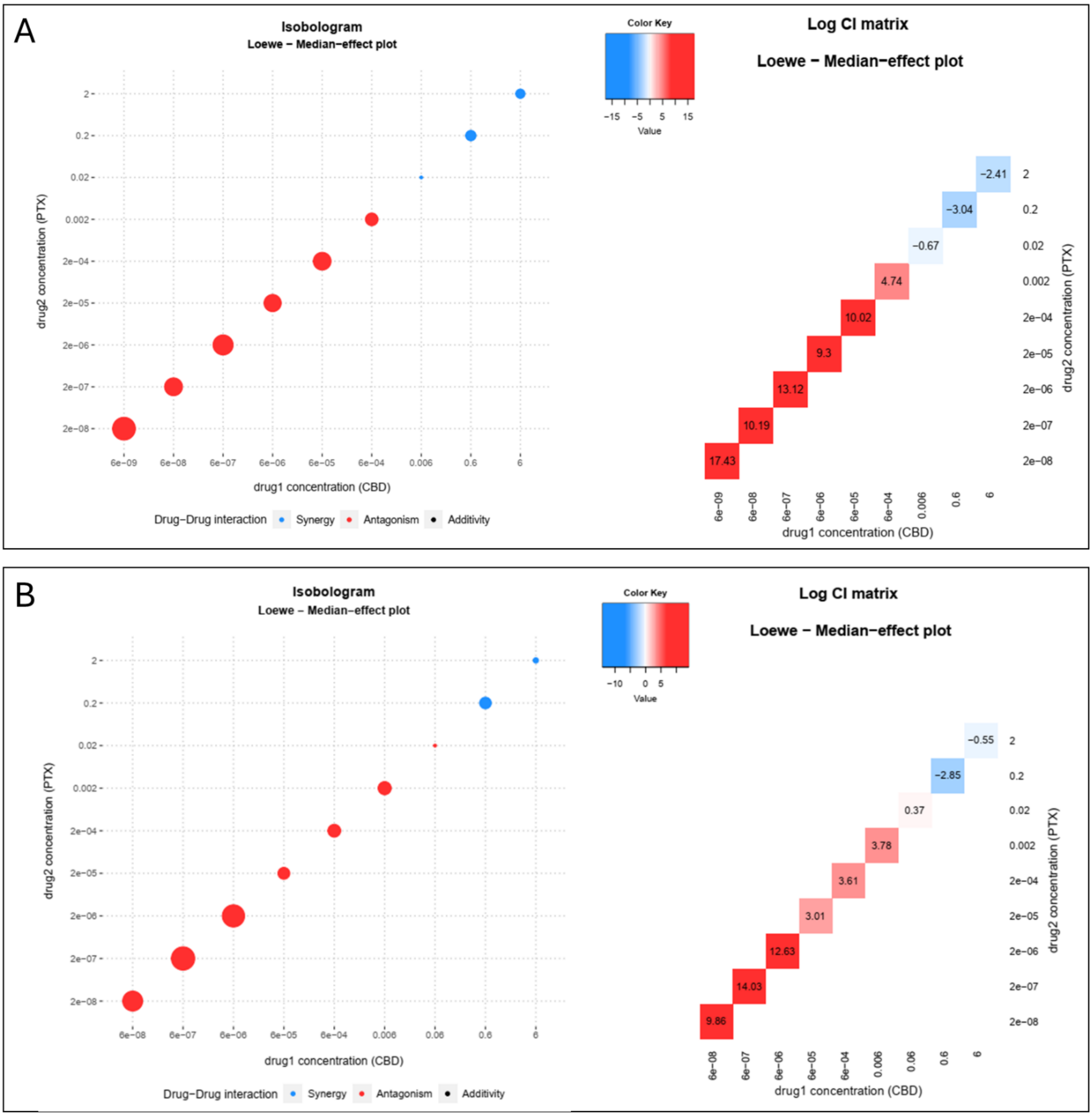

Figure S5 – Drug combination results in NLC-CBD-PTX obtained in SiCoDEA at: https://sicodea.shinyapps.io/shiny/. Isobologram and combination index (CI) graphs obtained with the results of cellular viability in 24 h (A) and 48 h (B) compared with commercial formulations.

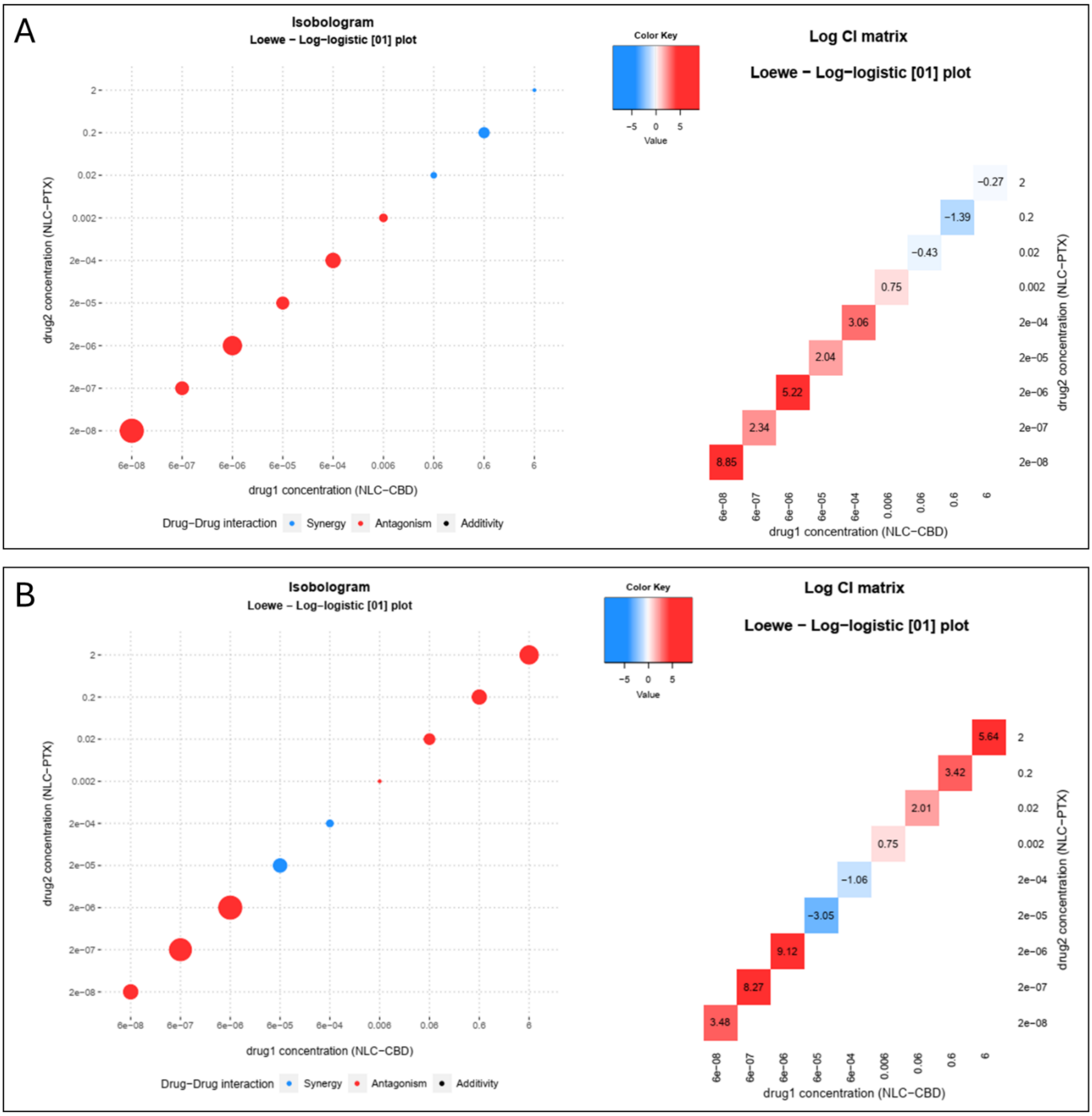

Figure S6 – Drug combination results in NLC-CBD-PTX obtained in SiCoDEA at: https://sicodea.shinyapps.io/shiny/. Isobologram and combination index (CI) graphs obtained with the results of cellular viability in 24 h (A) and 48 h (B) compared with NLC controls.

Table S1. ANOVA test for factorial model of the response: Size of nanoparticles.

**Source**	**Sum of Squares**	**df**	**Mean Square**	**F-value**	**p-value**	
**Model**	2352.32	7	336.05	17.46	0.0194	significant
A-MM	107.31	1	107.31	5.58	0.0993	
B-SPC	291.61	1	291.61	15.15	0.0301	
C-P68	1548.46	1	1548.46	80.47	0.0029	
AB	187.21	1	187.21	9.73	0.0525	
AC	40.95	1	40.95	2.13	0.2407	
BC	102.96	1	102.96	5.35	0.1037	
ABC	73.81	1	73.81	3.84	0.1451	
**Residual**	57.73	3	19.24			
Lack of Fit	0.4667	1	0.4667	0.0163	0.9101	not significant
Pure Error	57.26	2	28.63			
**Cor Total**	2410.05	10				

Table S2. ANOVA test for factorial model of the response: PDI of nanoparticles.

**Source**	**Sum of Squares**	**df**	**Mean Square**	**F-value**	**p-value**	
**Model**	0.0142	5	0.0028	35.56	0.0021	significant
A-MM	0.0029	1	0.0029	35.75	0.0039	
B-SPC	0.0004	1	0.0004	5.09	0.0870	
C-P68	0.0074	1	0.0074	92.58	0.0007	
AC	0.0013	1	0.0013	16.63	0.0151	
BC	0.0022	1	0.0022	27.73	0.0062	
Curvature	0.0026	1	0.0026	32.02	0.0048	
**Residual**	0.0003	4	0.0001			
Lack of Fit	0.0002	2	0.0001	1.93	0.3407	not significant
Pure Error	0.0001	2	0.0001			
**Cor Total**	0.0170	10				

Table S3. ANOVA test for factorial model of the response: Zeta potential of nanoparticles.

Source	Sum of Squares	df	Mean Square	F-value	p-value	
**Model**	180.33	5	36.07	28.38	0.0011	significant
B-SPC	129.20	1	129.20	101.67	0.0002	
C-P68	6.35	1	6.35	5.00	0.0756	
AC	2.39	1	2.39	1.88	0.2288	
BC	21.35	1	21.35	16.80	0.0094	
ABC	21.03	1	21.03	16.55	0.0097	
**Residual**	6.35	5	1.27			
Lack of Fit	2.96	3	0.9875	0.5824	0.6816	not significant
Pure Error	3.39	2	1.70			
**Cor Total**	186.68	10				

Table S4 - R^2 coefficients of mathematical models applied to the *in vitro* release kinetic curves (Figure 5), calculated using KinetDS 3.0 software.

				$\mathbf{R^2}$		
				Models		
	**Formulation**	**0 order**	**First order**	**Korsmeyer-Peppas**	**Weibull**	**Logarithm**
**PTX**	**Taxol**	0.7168	0.2943	0.7768	0.9004	0.9731
	**NLC-CBD-PTX**	0.9606	0.5395	0.9590	0.9710	0.8375
**CBD**	**CBD**	0.7504	0.4062	0.8715	0.9145	0.9371
	**NLC-CBD-PTX**	0.8983	0.5255	0.9579	0.9706	0.9206
